# Stem Water Storage Dynamics in Amazonian Palms and Dicotyledonous Trees

**DOI:** 10.1007/s12042-026-09478-9

**Published:** 2026-05-22

**Authors:** Lion R. Martius, Thaise Emilio, Thales Moreira de Lima, Pablo Sanchez-Martinez, Antonio C. L. da Costa, Maurizio Mencuccini, Patrick Meir

**Affiliations:** 1 School of GeoSciences, University of Edinburgh, King’s Buildings, Alexander Crum Brown Rd, Edinburgh, EH9 3FF UK; 2 Center for Research on Biodiversity Dynamics and Climate Change (CBioClima), São Paulo State University (UNESP), Institute of Biosciences, Campus Rio Claro, São Paulo, Brazil; 3 Royal Botanic Garden Edinburgh, Edinburgh, EH3 5LR UK; 4 Institute of Ecology and Evolution, University of Edinburgh, Charlotte Auerbach Road, Edinburgh, EH9 3FL UK; 5 Museu Paraense Emílio Goeldi , Campus de Pesquisa, Av. Perimetral, Belém, PA 66077- 830 Brazil; 6 Instituto de Geociências, Universidade Federal do Pará, Belém, PA 66075-110 Brazil; 7 CREAF, Campus UAB, Cerdanyola del Vallés, 08193 Spain; 8 ICREA, Barcelona, 08193 Spain

**Keywords:** Frequency-domain reflectometry, Ecophysiology, Drought, Stem water content, Palms, Amazon rainforest

## Abstract

Stem water storage plays a key role in buffering plants against drought, yet continuous measurements of stem water content remain rare in tropical forest trees. Using frequency domain reflectometry (FDR) sensors, we continuously monitored stem water storage and mobilisation dynamics in co-occurring palm and dicotyledonous tree species in the eastern Amazon during the extreme 2023 drought. This approach provides, to our knowledge, the first temporal high-resolution dataset of stem hydration in Amazonian palms (*Astrocaryum vulgare* Mart., *Oenocarpus distichus* Mart.) and co-occurring trees. Palms demonstrated substantially higher absolute water storage capacity and greater seasonal and diurnal water mobilisation compared to dicots. Using a novel analysis for critical relative stem water content thresholds revealed striking physiological divergence: palms maintained high relative diurnal discharge capacity, (i.e., the amount of water that can be released from the stem during the day, relative to the maximum capacity), at hydration levels where dicots exhibited significant impairment. In addition, we identify a common soil moisture threshold at 0.19 m^3^ m^−3^ below which stem water declines rapidly, indicating likely hydraulic disconnection from the soil. We demonstrate the usefulness of FDR technology combined with a new threshold analysis to describe how tropical palms and trees respond to increasing drought stress under climate change.

**Supplementary Information:**

The online version contains supplementary material available at 10.1007/s12042-026-09478-9.

## Introduction

Palms (Palmae/Arecaceae) are one of the most abundant, diverse and economically significant family of plants within the Neotropics (Muscarella et al. 2020; Martins et al. 2024). Despite this prominence, their global distribution is almost entirely confined to narrow geographical and climatic limits within the tropics, with a marked decline in both species richness and abundance at higher elevations and absolute latitudes (Eiserhardt et al. 2011; Reichgelt et al. 2018; de Lima et al. 2022). In Amazonian forests, six out of the ten most dominant tree species belong to the Arecaceae family, which can account for up to 60% of total basal area in some Neotropical forest types (ter Steege et al. 2013; Cooper et al. 2024). Palms present a principal feature of tropical forest structure in lowland floodplain, *terra-firme* and swamp forests and are integral to the biomass, biogeochemical and hydrological cycling at ecosystem scale (Emilio et al. 2014; Salm et al. 2015; Dargie et al. 2017; Dalagnol et al. 2022; Amaral et al. 2025; Verona et al. 2026).

The strict climatic constraints on the biogeography of palms were recognised in the 19th century by Carl F. P. von Martius in his monumental *Historia Naturalis Palmarum*. In the chapter *De Palmarum Rationibus Geographicis*, Martius wrote: “The dignity of palm trees in phytogeography is supreme […], on which physiologists should focus their attention, so that […] they may investigate both the subtle changes in climate and their influence and causes on vegetation.” (Martius et al. 1823).

Despite Martius’ early call for physiological research, palms have remained largely peripheral in studies of ecophysiology and plant hydraulics, despite or perhaps due to their anatomical and morphological uniqueness (Tomlinson 2006; Renninger and Phillips 2016). However, palms could prove to be useful model organisms to answer fundamental questions in plant hydraulics. The organisation of their stems is relatively simple. Parenchyma forms the main component, and vascular bundles are distributed within, but as monocots they lack vascular cambium (Tomlinson 2006). While xylem and phloem tissues in palms share similar cell types as in dicotyledonous angiosperms, vessel elements in palms are broader than in dicots, making palms highly efficient in terms of water transport under well-hydrated conditions (Renninger et al. 2010, 2013; Kunert et al. 2015; Aparecido et al. 2015). However, palms lack secondary growth, meaning that vascular bundles have to maintain their conductive ability throughout the plant’s life, which can well surpass 100 years in some specimens, while also reaching heights of up to > 60 m (Tomlinson 2006; Renninger and Phillips 2016). This raises important questions for our fundamental understanding of embolism resistance in plants and its relationship with vessel diameter (Emilio et al. 2019), the potential of xylem refilling mechanisms (Klein et al. 2018), and the involvement of parenchymatous tissue in this process (Ny et al. 2021; Słupianek et al. 2021; Kawai et al. 2022). Despite their abundance and ecological importance, and while ecophysiological studies of woody plants continue to be biased towards dicotyledonous trees, our understanding of how palms respond to drought and climate change—particularly how they store and regulate water —remains limited (Portela et al. 2020; Emilio et al. 2021).

Recent years have seen growing interest among ecophysiological studies in the role of internal water storage as a buffer against drought stress in trees within ecophysiological studies (Salomón et al. 2017; Martinez-Vilalta et al. 2019; Costa et al. 2023b). Stem water storage and hydraulic capacitance, the capacity of plant tissues to store and release water per unit water potential difference, plays a key role in mitigating short-term water deficits in trees, particularly under high atmospheric demand (Richards et al. 2014; Salomón et al. 2017). In this context, the ability of palms to store large quantities of water needs to be highlighted (Martius et al. 1823, 2024). The high proportion of living parenchyma cells suggests a potentially large internal reservoir of mobile water. These characteristics have led to the hypothesis that palms may function as hydraulic capacitors, maintaining leaf water supply even under declining soil moisture and increasing vapor pressure deficit. However, direct measurements of water content dynamics in palm stems remain scarce. Partly due to uncertainties in sensor calibration, usage of water content sensors has been limited. Continuous measurements of water content in palms are particularly scarce (but see Holbrook and Sinclair 1992; Sperling et al. 2015), and no such work has been ever undertaken in Amazonian palms. Yet this knowledge is particularly urgent given the increasing frequency and intensity of extreme drought and heat in Amazonia (Marengo et al. 2024). Studies have suggested that palm communities have remained remarkably unchanged in recent decades in more climatically stable regions, such as western Amazonia (Olivares et al. 2017). In central Amazonia, palm community responses to severe drought depend strongly on the amount of water received in previous wet periods (Rodrigues-Filho et al. 2024). Stem water storage may contribute to this “insurance effect” that buffers community stability under increasing climatic variability. Understanding the thresholds of palm resilience to drought, and for how long internal stem water storage can sustain them, is therefore critical for predicting the future of this dominant life-form in Amazonian forests.

To overcome methodological challenges, recent work has established a widely applicable general calibration for the use of stem water sensors in woody tissue for both dicotyledonous trees and monocotyledonous palms (Martius et al. 2024). Frequency domain reflectometry (FDR) technology can provide a low-cost and minimally invasive approach to measure volumetric stem water content. FDR sensors detect changes in the dielectric permittivity of plant tissue—a property that varies with water content. Given the large water content of palm stems, this method may be particularly well-suited to capturing subtle changes in tissue hydration over time and under the influence of climate, especially during the recent El Niño 2023/24, which triggered one of the most severe droughts on record across the Amazon (Espinoza et al. 2024).

Here, we present a study using FDR technology to investigate stem water content dynamics in two Amazonian palms, *Astrocaryum vulgare* Mart. and *Oenocarpus distichus* Mart. and co-occurring dicotyledonous trees, during a seasonal dry-down in a well-drained *terra-firme* forest in eastern Amazonia. We examine how stem hydration fluctuates on both diurnal and seasonal timescales, and how these changes correspond to soil moisture and precipitation. We compared the palms with five co-occurring dicot trees growing below the canopy, chosen to reflect a gradient of wood density and varying hydraulic traits identified in previous studies from this study site (Negrão-Rodrigues et al. 2025). This is the first study to report in vivo, high-resolution stem hydration data in Amazonian palms. Our aims were to assess the hydraulic strategies of palms and co-occurring dicots by: (i) quantifying seasonal and diurnal stem water content dynamics using continuous FDR monitoring during the 2023 dry season; (ii) determining critical hydration thresholds and their impact on relative diurnal discharge capacity; and (iii) characterising soil-stem water content relationships. Based on palm anatomical structure, we hypothesised that these monocots in contrast to co-occurring dicots: (1) demonstrate substantially greater absolute water storage and mobilisation capacity; (2) show superior drought buffering, allowing them to mobilise water under lower relative stem hydration levels; and (3) demonstrate stronger decoupling from soil moisture availability during seasonal water deficits. A key goal of this study is to demonstrate the value of FDR sensing for palm ecophysiology and drought impacts and to offer first insights into stem water content dynamics in palms specifically in response to events of extreme drought.

## Materials and Methods

### Study Site, Species Sampling and Meteorology

The study was conducted in an Amazonian lowland tropical rainforest situated in *Floresta Nacional de Caxiuanã*, state of Pará, in northern Brazil (1°43′S, 51°27′W). The site is designated as an old-growth *terra-firme* forest and is located within Xingu–Tocantins–Araguaia moist forests ecoregion. The seasonally dry evergreen forest receives an annual precipitation of between 2,000 and 2,500 mm, with monthly rainfall dropping below < 100 mm month^− 1^ during the dry season (August—November). The equatorial climate results in steady temperatures throughout the year with a mean below-canopy temperature of ~ 26 °C (Meir et al. 2015). The physiological study was conducted in two monocotyledonous palm individuals, *Astrocaryum vulgare* Mart. and *Oenocarpus distichus* Mart., and five dicotyledonous trees, namely *Protium tenuifolium* (Engl.) Engl., *Vouacapoua americana* Aubl., *Licania octandra* (Hoffmanns. ex Schult.) Kuntze, and two individuals of *Pouteria decorticans* T.D.Penn, to cover a wide range of wood densities, presented in the result table (Table 1), and hydraulic traits (Negrão-Rodrigues et al. 2025). We excluded large emergent trees from our analysis to minimise the impact of microclimate, as the palms from this study were growing under the canopy, reflecting a subcanopy leaflet economy (Oda et al. 2023). The measurement period (163 days) lasted from the end of the wet-season in July throughout the entire El Niño drought, capturing the start of the following wet season until December 2023 (Martius et al. 2025).

The plot was equipped with a meteorological station. We measured data on precipitation, air temperature (T [°C]) and relative humidity (RH in %). Sensors for T and RH were installed in the subcanopy (16 m). Vapour pressure deficit (VPD) was calculated using T and RH using bigleaf package in R (Knauer et al. 2018). Soil water content was measured in soil pits at three different depths at an hourly resolution at 100 cm, 250 cm and 400 cm respectively using TDR sensors (CS616, Campbell Scientific), and precipitation was monitored using a tipping bucket rain gauge (TE525MM, Campbell Scientific).

### Stem Water Content Measurement

Frequency-domain reflectometry sensors (Teros 12, Meter Group, Pullman, WA, USA) were installed in the southward-facing site of the trunks at breast height of the aforementioned individuals, measuring at a resolution of 15 min. These three pin sensors measure dielectric permittivity (ε_stem_) and temperature (T_stem_ [°C]) within the stem. We carefully removed the bark of the dicotyledonous trees, to make sure the sensors were situated within the sapwood. Naturally, palms lack vascular and cork cambium, and sensors were directly placed into the epidermis. Three parallel holes were drilled into the stems making sure that the drill bits were the same length as the sensor needles. To maximise accuracy during this process, we used a drill guide. The 3-mm drill bit was slightly thinner than the sensor needles (3.175 mm), assuring close contact between the sensor and the woody tissue in order to avoid air contact and related xylem dehydration (Nakada 2025). Once the sensors were in place, they were sealed using a silicon-based sealant and protected using environmental radiation shields. For a detailed description of the installation protocol, see https://github.com/lionmartius/Splish-Splash-Sap. Shortened (30 mm length) sensors were installed in July 2023. Volumetric water content ($\:{\theta\:}_{stem}$) was accurately estimated from its relationship with $\:\sqrt{{\epsilon\:}_{stem}}$ based on laboratory-derived calibration at 25 °C (Martius et al. 2024):

  

1 $$\:{\theta\:}_{stem}=0.2227\:\sqrt{{\epsilon\:}_{stem}}-0.396$$

However, the dielectric constant is inversely proportional to temperature, meaning it decreases with increasing temperature, due to the reduced polarisation capability of molecules, with increased thermal motion, making temperature calibrations of these sensors necessary. Temperature (T_stem_) corrected water content ($\:{\theta\:}_{T}$ ) was estimated using an empirical correction:

  

2 $$\:{\theta\:}_{T}={\theta\:}_{stem}+{{\Delta\:}\mathrm{T}}_{stem}\mathrm{*}{\upbeta\:}$$

where $\:{\Delta\:}\mathrm{T}$ is the difference between the measured T_stem_ and reference T_ref_ $\left(25^\circ C\right)$and $\:{\upbeta\:}$ is the temperature effect (slope coefficient: −0.000974).

### Quantification of Stem Water Storage and Temporal Variability

Maximum turgid stem water content (θ_t_) was estimated as the seasonal maximum (99^th^ percentile) volumetric water content for each individual. Seasonal water loss magnitude (seasonal Δθ_stem_) was calculated as the difference between θ_t_ and minimum (1^st^ percentile) seasonal water content (θ_d_). The diurnal magnitude, which is the diurnal maximum discharge capacity was computed as the 99^th^ percentile of daily water content ranges (diurnal Δθ_stem_) across the study period for each individual. Relative stem water content (RWC, dimensionless between 0 and 1) was calculated as the ratio of observed volumetric water content relative to tree-specific θ_t_ values. The daily maximum RWC (night-time) served as a proxy for daily hydration status. The diurnal discharge capacity was quantified as the percentage of diurnal Δθ_stem_ relative to maximum observed amplitude for each individual. Additionally, we estimated the total storage (*S*) of active water (i.e., the physiologically active part of the stem water, which is stored within the sapwood of dicotyledonous trees and entire stem for palms). For palms we assumed a perfect cylinder and calculated the stem volume by the volumetric water content similar to Sperling et al. (2015). For trees, we calculated *S* for the sapwood only, by multiplying the sapwood volume by the volumetric stem water content. We estimated the sapwood volume, using an established allometric relationship between DBH and sapwood depth estimated for Amazonian trees from the literature (Aparecido et al. 2019), and estimated the tapered sapwood volume (V_S_) for trees by multiplying the sapwood area (*A*_*s*_) with the height (*H*) and the tapering factor (*f*) of 0.5 taken from the literature (Cannell 1984), where ${\mathrm V}_s=f\cdot A_s\cdot H$. Heights were measured in the field using a Forestry Pro II Laser Rangefinder (Nikon Corporation, Tokyo, Japan), and DBH using a measuring tape.

### Critical Relative Stem Water Content Analysis

Critical RWC thresholds (RWC_crit_), where relative stem hydration leads to a critical reduction in diurnal stem water discharge capacity, were determined using the relationship between daily relative discharge capacity, computed as diurnal Δθ_stem_/ 99^th^ Δθ_stem_ and daily max stem RWC. Critical reductions in diurnal stem water discharge are interpreted as hydraulic strain, i.e., the physiological deviation from optimal functioning, induced by drought as the external stress. Accordingly, trees were classified into levels of hydraulic strain under environmental drought stress as follows: moderate strain when diurnal discharge from the stem dropped to 10–20%, high strain to 5–10%, and severe strain below 5% of its maximum discharge capacity. These categories of hydraulic strain describe the tree-specific magnitude of functional response in stem water dynamics (i.e., the deviation from physiological optima) in response to drought stress. For the threshold analysis, we concentrated on the proportion of the curve with stem RWC < 0.95, which contains the values when significant water stress occurred. We calculated RWC_crit_ as the mean RWC during periods of high strain (< 10% of diurnal discharge capacity) for each individual, representing the hydration level where diurnal discharge becomes critically impaired. The RWC at the point of maximum release capacity (RWC_mrc_) per individual tree was calculated as the mean RWC where daily discharge is larger than 75% of its maximum capacity, corresponding to near-optimal hydraulic functioning and minimal strain. This formulation explicitly distinguishes between externally imposed drought stress and internally expressed hydraulic strain, enabling a mechanistic and plant specific interpretation of stem water dynamics under water limitation (Rosado et al. 2023).

To determine whether reductions in the diurnal discharge capacity from the stem of individual trees during drought stress (where water availability drops below RWC ≤ 0.95) are primarily influenced by atmospheric demand rather than by plant hydraulic regulation (e.g., stomatal regulation), we analysed Pearson correlations between daily maximum VPD and relative diurnal discharge capacity during drought stress. Strong positive correlations between VPD and diurnal discharge capacity would indicate increased transpiration under higher evaporative demand. However, weak or negative correlation would suggest that plant physiological regulation, such as stomatal closure occurs during drought stress (soil moisture deficit), controlling discharge capacity independently of atmospheric conditions. Since correlations between VPD and amplitude reduction across all individuals were weak and negative (palm mean *r* = − 0.146 ± 0.159; dicot mean *r* = − 0.297 ± 0.124), we conclude that reductions in diurnal discharge capacity during stress are indeed plant-regulated responses to drought stress (e.g., stomatal control), rather than inhibited by a highly humid (non-demanding) atmosphere (Supplementary Information Fig. S1).

### Soil Moisture Threshold Responses

Generalized additive mixed models (GAMs) were fitted using the mgcv package in R, with separate smooth functions for the daily maximum soil volumetric water content for each plant type to capture non-linear relationships between soil- and stem water content. First derivatives of the fitted GAM curves were calculated to identify rapid change and to quantify the magnitude of stem water content responses to soil moisture variation. We estimated the soil-moisture threshold for the rapid decline in stem water reserves from the pooled GAM by computing the first derivative of the smooth and its confidence interval across the observed soil volumetric water-content range. The threshold was defined as the first soil water-content value where the derivative indicated a significant and pronounced decline (upper confidence limit below zero and derivative within the strongest negative quartile), sustained for at least five consecutive evaluation points. All analyses used REML smoothing parameter estimation with basis dimension k = 6 to balance model flexibility and overfitting prevention. In addition to the volumetric soil moisture, we calculated soil relative water content using 99^th^ percentile normalisation from the long-term soil moisture dataset in Caxiuanã (Meir et al. 2015; Sanchez-Martinez et al. 2025).

### Data Analysis

Statistical differences between the total, seasonal and diurnal stem water content between palms and dicots were assessed using one-sample t-tests, which evaluated whether dicot values differed significantly from palm group means. In addition, we conducted non-parametric permutation tests (5000 iterations), based on the difference in group means and suitable for small sample sizes (*n* = 2 palms, *n* = 5 dicots). A null distribution was generated by randomly permuting group labels, and two-sided p-values were calculated as the proportion of permuted differences exceeding the observed difference in absolute value. Since the results from both tests converged, t-tests results were presented.

Due to the limited data on palms from this study, we supplemented our analysis with additional stem water content data from an Atlantic Rainforest site (Parque Estadual de Porto Ferreira, 21° 50’ 41” S, 47° 25’ 25” W). This dataset, drawn from a global database of stem water content currently under preparation (Buranelli and Martius 2025), employed an identical experimental setup as used in our Amazonian study. FDR sensors were installed in eight palms (four *Syagrus romanzoffiana* (Cham.) Glassman and four *Euterpe edulis* Mart.) and four dicotyledonous trees (four *Protium* spp.), with stem water content measured from 05th of November until 01st of December 2025 during the rainy season (Supplementary Information Fig. S2). Since the Atlantic Forest trees were not subjected to drought conditions, this dataset was not integrated into the drought stress analyses central to our study. Instead, it was solely used to provide complementary evidence on inherent differences in total stem water storage capacity between palms and dicotyledonous trees. To test for significant differences between palm and dicotyledonous tree response curves in the GAM soil threshold analysis, we performed model comparison using ANOVA. All statistical analyses were performed in R (version 4.3.0) (R Core Team 2024).

## Results

### Stem Water Storage Capacity and Temporal Variability

**Fig. 1:**
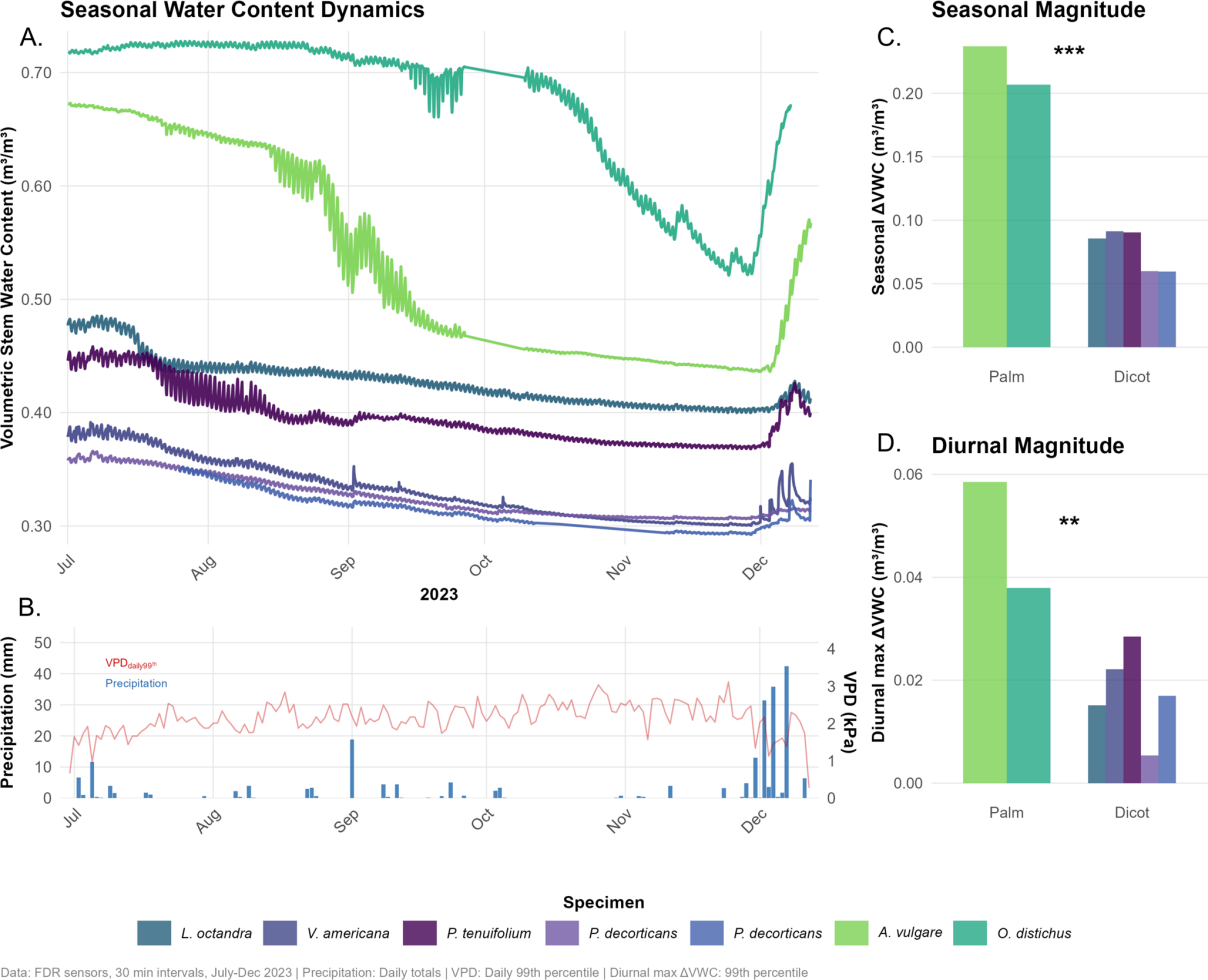
**A**) Temporal stem water content (m^3^ m^–3^) dynamics of two palms and five dicotyledonous trees, measured during a seasonal dry-down between July and December 2023 in the eastern Amazon. **B**) Maximum (99^th^) vapour pressure deficit (VPD) and daily precipitation data was plotted below the stem water content data. **C**) The seasonal magnitude was calculated as the difference of individual tree level turgid (99^th^ percentile) stem water content and their seasonal minimum (1^st^ percentile). **D**)The diurnal magnitude was calculated as the maximum (99^th^ quantile) daily stem water content difference between daily minimum (1^st^) and maximum (99^th^). Stem water content was measured using FDR technology and calibrated according to Martius et al. (2024)

Our study on stem water content dynamics across seven Amazon Forest species revealed substantial differences between palms and dicots across all measured variables. Despite modest sample sizes (*n* = 2 palms, *n* = 5 dicots), both permutation tests and one-sample t-tests consistently detected statistically significant differences. Palms demonstrated markedly higher maximum turgid stem water content (θ_t_) compared to dicots (0.700 ± 0.039 vs. 0.411 ± 0.058 m³ m^−^³, respectively), representing a 70% larger absolute storage capacity (Fig. 1; Table 1). This difference was statistically significant (one-sample t-test *p* < 0.001), indicating substantial biological relevance. Furthermore, additional data from eight palms and four dicotyledonous trees collected during the rainy season at an Atlantic Forest site (θ_mean_ 0.590 ± 0.070 m^3^m^− 3^ vs. 0.380 ± 0.030 m^3^m^− 3^ respectively; t = 5.9, df = 10, *p* < 0.001) further support our findings of large water storage capacity of tropical palms. This reinforces that the higher stem water content observed in palms is a consistent pattern across arborescent palms beyond our initial Amazonian sample and is unlikely to result from limited sampling. In addition, we estimated the total storage capacity of active water (*S*; i.e., water that is physiologically available and dynamically involved in daily water transport and storage), and estimated that the 20 m tall (and a DBH of 0.22 m) *A. vulgare* stores 0.53 m^3^ (530 L) of active water in its stem, and 0.36 m^3^ (360 L) for the smaller 13 m tall (and a DBH of 0.22 m) *O. distichus*. A dicotyledonous tree of the same size as *A. vulgare* (h = 20 m and DBH = 0.22 m, sapwood depth = 3.5 cm) would store just 0.086 m^3^ (86 L) of active water in its sapwood.

**Table 1:** Stem water content parameters and wood density (g cm^− 3^) for two Amazonian palm and five dicot individuals. θ_t_ represents maximum (99^th^) turgid volumetric water content (m³ m⁻³), θ_d_ represents dry (minimum, 0.01st) volumetric water content (m³ m⁻³), and θ_mean_ represents seasonal mean volumetric water content ± standard deviation (m³ m⁻³). CV % is the coefficient of variation expressed as percentage. Water deficit % represents the percentage water loss relative to maximum turgid content ((θ_t_ - θ_d_)/θ_t_ × 100). Absolute water loss is calculated as θ_t_ - θ_d_ (m³ m⁻³). Max diurnal discharge capacity (99^th^ percentile) represents the maximum daily water content fluctuation (m³ m⁻³) observed during the study period

Specimen	θ_t_	θ_d_	θ_mean_	CV %	Water deficit %	Absolute water loss	Max diurnal discharge capacity	WD
*Astrocaryum vulgare*	0.673	0.436	0.532 ± 0.089	16.7	35.2%	0.237	0.058	0.63 ± 0.21
*Oenocarpus distichus*	0.728	0.521	0.674 ± 0.063	9.38	28.4%	0.207	0.038	0.29 ± 0.07
*Protium tenuifolium*	0.459	0.367	0.397 ± 0.025	6.41	20.1%	0.092	0.028	0.54 ± 0.03
*Vouacapoua americana*	0.391	0.300	0.333 ± 0.026	7.88	23.3%	0.091	0.025	0.82 ± 0.05
*Licania octandra*	0.485	0.400	0.427 ± 0.021	5.0	17.5%	0.085	0.012	0.69 ± 0.05
*Pouteria decorticans*	0.366	0.306	0.326 ± 0.018	5.6	16.4%	0.060	0.016	0.75 ± 0.12
*Pouteria decorticans*	0.352	0.292	0.315 ± 0.016	5.1	17.0%	0.060	0.013	0.75 ± 0.12

Palms exhibited nearly three times the absolute seasonal volumetric stem water discharge (seasonal Δθ_stem_) compared to dicots (0.222 ± 0.021 vs. 0.077 ± 0.016 m³ m^−^³). This difference was significant (one-sample t-test *p* < 0.001), indicating that palms possess substantially greater capacity for seasonal stem water depletion than dicots (Fig. 1). Furthermore, the diurnal maximum (99^th^ quantile) water content magnitude (diurnal Δθ_stem_) was more than twice as large in palms compared to dicots (0.048 ± 0.015 vs. 0.018 ± 0.009 m^3^ m^–3^), with a significant difference (one-sample t-test *p* = 0.009), representing an enhanced capacity and available potential water pool in palms for rapid daily water mobilisation from the stem (Fig. 1; Table 1).

### The Relationship Between Stem Hydration and Diurnal Water Discharge

**Fig. 2:**
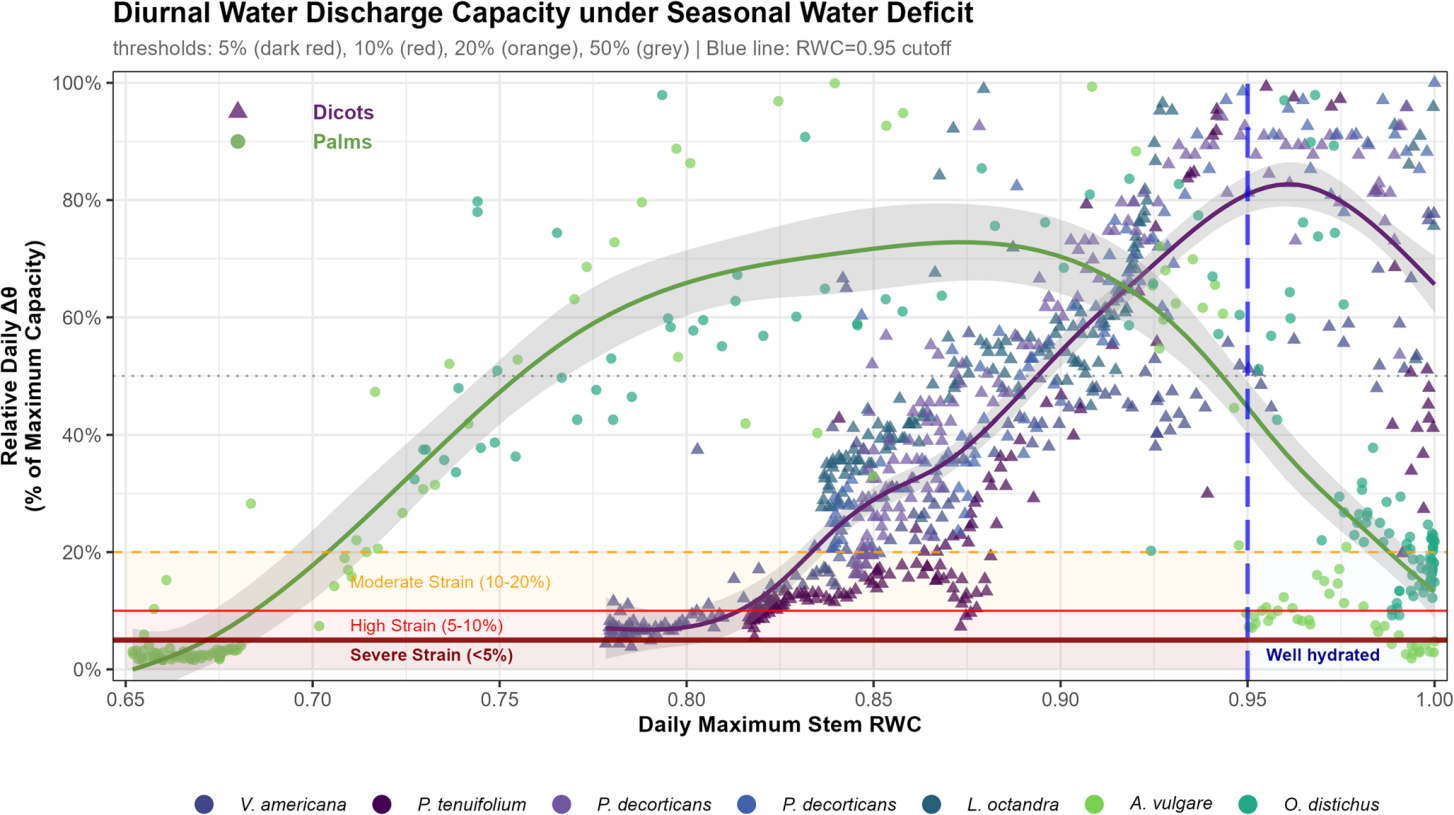
Daily maximum relative stem water content (RWC) as a function of relative diurnal water discharge capacity (Δ0) expressed as percentage of individual maximum capacity (99^th^ percentile) for two palm species (circles: *Astrocaryum vulgare* in green, *Oenocarpus distichus* in teal) and five dicotyledonous tree species (triangles). Coloured trend lines show species-level GAM fits with 95% confidence intervals. Horizontal lines indicate strain thresholds: severe strain below 5% capacity (dark red solid), high strain 5-10% capacity (red solid), moderate strain 10-20% capacity (orange dashed), and 50% capacity reference (grey dotted). Coloured shaded zones highlight strain regions for RWC ≤ 0.95. The vertical blue dashed line at RWC = 0.95 indicates the threshold above which strain analysis was excluded due to soil water availability rather than hydraulic limitations on stem water discharge capacity. Points represent individual daily measurements during the 2023 monitoring period. Stem water content was measured using FDR technology and calibrated according to Martius et al. (2024)

The relationship between daily maximum relative stem water content (RWC), indicating daily tree hydration status, and diurnal stem water discharge capacity, indicating plant regulated diurnal depletion and refilling capacity, revealed distinct physiological strategies and thresholds between palms and dicots during seasonally increasing water deficit (Fig. 2). All species demonstrated a strong positive relationship between stem water availability (where RWC < 0.95, excluding days with high water abundance) and diurnal discharge capacity, but with markedly different critical thresholds and hydraulic strain. 

Critical RWC (RWC_crit_) thresholds, where relative stem hydration leads to reduced diurnal discharge capacity reaching critical low functional levels (< 10%), were markedly lower in palms (0.67 ± 0.01) than in dicots (0.81 ± 0.03; Fig. 2; Table 2). The RWC at the point of maximum release capacity (RWC_mrc_) values, calculated from the entire dataset including high-hydration periods showed that palms achieve maximum diurnal discharge capacity at lower hydration levels (0.87 ± 0.02) compared to dicots (0.94 ± 0.01) This fundamental difference means that palms exhibit high levels of diurnal stem water content dynamics at RWC values where dicots already show significant physiological impairment (Fig. 2; Table 2).

Drought strain intensity patterns revealed species-level vulnerabilities during the study period. *Astrocaryum vulgare* experienced the most severe strain with 67 days falling below critical strain thresholds (≤ 10% water discharge capacity), out of which 65 days fell below severe strain thresholds (≤ 5% water discharge capacity), indicating prolonged and abrupt decrease in water discharge capacity. *Oenocarpus distichus* on the other hand exhibited exceptional drought tolerance, never approaching critical thresholds and maintaining high relative diurnal discharge capacity throughout the seasonal cycle. Among dicots, stress tolerance varied. *Vouacapoua americana* suffered 46 days below critical thresholds but only 2 days below the severe strain threshold, while *P. tenuifolium* showed a conservative strategy with 70 days under moderate strain (10–20% capacity) but avoiding critical strain conditions. The two *P. decorticans* individuals and *L. octandra* exhibited little strain, with few days falling in the moderate category (1, 16, 1 days respectively), suggesting species-level strategies for drought avoidance versus tolerance within the dicot group. Remarkably, palms demonstrated greater tolerance to low stem RWC conditions, maintaining functional diurnal water discharge at hydration levels that severely compromise dicot physiology, indicating fundamentally different hydraulic strategies and drought adaptation mechanisms between these plant functional groups (Fig. 2).

**Table 2:** Physiological strain thresholds and drought exposure for seven Amazonian Forest trees. RWC_crit_ indicates the critical relative water content threshold where diurnal discharge capacity drops below 10% of maximum capacity (mean ± standard deviation). RWC_mrc_ represents the point of RWC at maximum release capacity, where the discharge capacity is at its inflection point (mean ± standard deviation). RWC_min_ represents the minimum observed relative water content during the study period. d_crit_ shows the number of days spent below critical thresholds, with values in parentheses indicating days below 10% and 5% discharge capacity, respectively. d_mod_ represents days spent under moderate strain conditions (10–20% discharge capacity). All RWC values are dimensionless ratios, and day counts reflect the 2023 monitoring period excluding days with RWC > 0.95 from strain analysis

Specimen	group	RWC_mrc_	RWC_crit_	RWC_min_	d_crit (10%,5%)_	d_mod (20%)_
*Astrocaryum vulgare*	Palm	0.86 ± 0.10	0.68 ± 0.03	0.65	67 (2,65)	6
*Oenocarpus distichus*	Palm	0.87 ± 0.09	< 0.73	0.73	0 (0,0,0)	0
*Protium tenuifolium*	Dicot	0.92 ± 0.05	0.81 ± 0.02	0.80	19 (19, 0)	71
*Vouacapoua americana*	Dicot	0.91 ± 0.05	0.78 ± 0.01	0.77	46 (42, 4)	24
*Licania octandra*	Dicot	0.94 ± 0.04	< 0.84	0.84	0 (0,0)	2
*Pouteria decorticans*	Dicot	0.94 ± 0.04	< 0.84	0.84	0 (0,0)	18
*Pouteria decorticans*	Dicot	0.95 ± 0.03	< 0.82	0.82	0 (0,0)	15

### Soil Moisture Threshold Responses

**Fig. 3:**
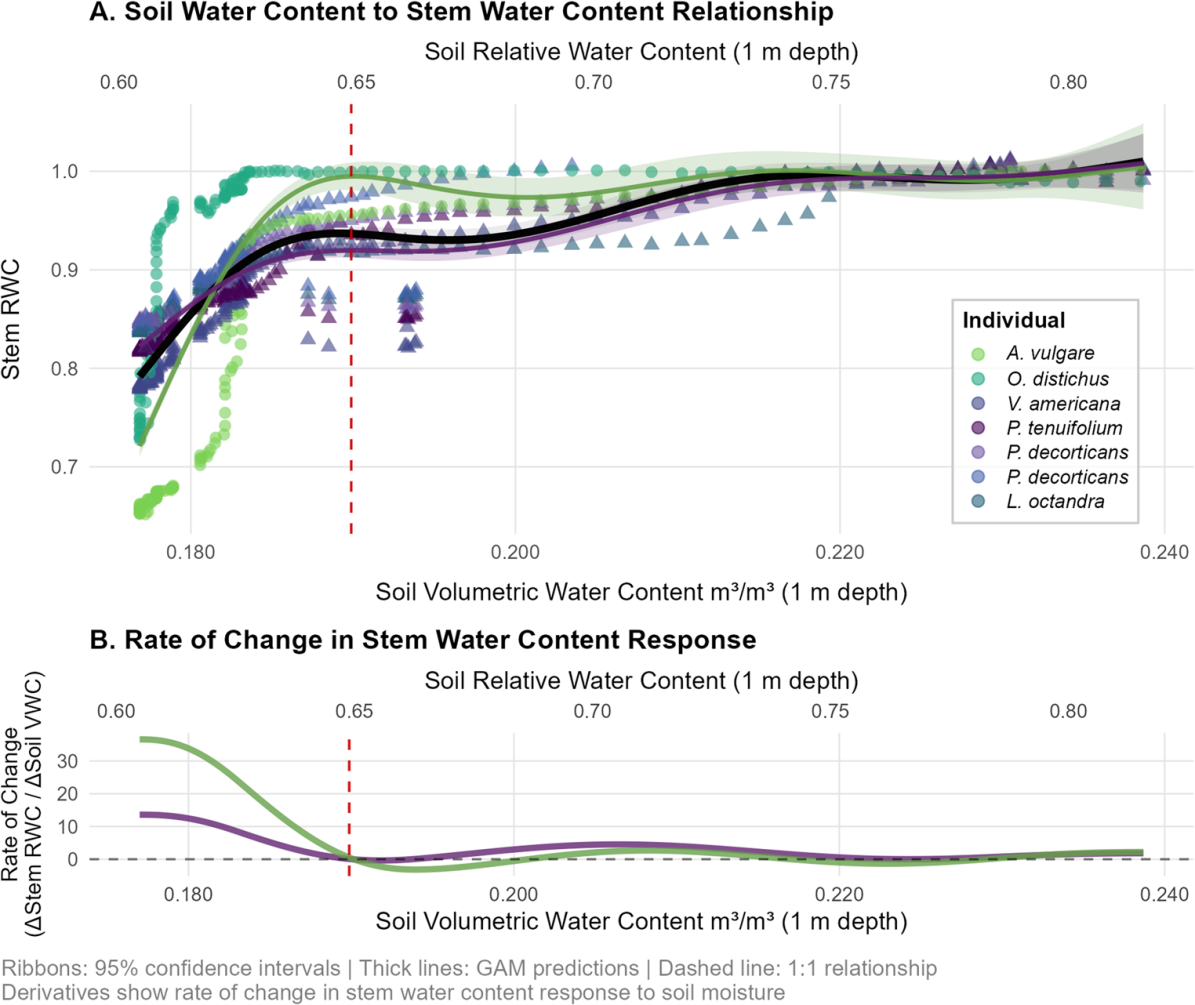
Non-linear relationship between volumetric soil water content (m^3^ m^–3^; measured at 1 m depth; primary x-axis at the bottom) and relative stem water content (RWC). The secondary x-axis (top x-axis) displays the relative soil moisture, which represents the soil water content divided by its long-term maximum (Meir et al. 2015). (**A**) Generalized additive model (GAM) analysis of daily maximum soil water content versus daily maximum stem RWC for seven individual Amazonian trees and palms. The GAM models used REML smoothing parameter estimation with basis dimension k=6 to balance model flexibility and overfitting prevention. Points represent individual daily observations coloured by specimen ID. Smooth lines show GAM predictions for each plant type (palms: green; dicotyledonous trees: purple) with 95% confidence intervals (shaded ribbons). The black line represents the smooth pooled across all trees. The red dashed vertical line marks the estimated soil-water threshold where the curve enters a sustained rapid-decline zone in stem RWC, indicating a coordinated response consistent with rapid stem water depletion and progressive hydraulic disconnection from the soil. (**B**) Rate of change analysis (first derivatives) quantifies the magnitude of stem water content response to soil moisture variation, plotted by plant-type. The red dashed line marks the threshold of 0.19 m^3^ m^–3^ of volumetric soil water content (~0.65 soil RWC), from which stem water sees a rapid decline across plant groups. Stem water content was measured using FDR technology and calibrated according to Martius et al. (2024)

Generalized additive mixed model (GAM) analysis revealed that all trees and palms maintained relatively stable stem water content with declining soil moisture (R^2^ = 0.35), showing a relatively steady response until a critical soil moisture threshold of 0.19 m³ m⁻³ (at 1 m depth; corresponding to ~ 0.65 RWC) was crossed, after which stem water content declined rapidly (Fig. 3). Nevertheless, stem water responses to the declining soil moisture differed between dicots and palms. Derivative analysis quantified the magnitude of these differential responses during water loss, revealing the range and intensity of stem water content changes across the soil moisture gradient. Palm derivatives ranged from + 36.61 to -3.14 (ΔStem RWC / ΔSoil VWC), while dicot derivatives ranged from + 13.61 to -0.37, indicating much more pronounced stem water loss when soil moisture crossed critical thresholds in Palms (Fig. 3b). Palms exhibited sharp transitions once thresholds were breached, while dicots showed a much more moderate, consistent slope throughout the soil moisture range.

## Discussion

Our findings provide the first high resolution measurements of stem water content dynamics in Amazonian palms, revealing fundamental differences in hydraulic strategies between these arborescent monocots and co-occurring dicotyledonous trees. The substantially higher absolute water storage capacity in palms supports the hypothesis that their parenchyma-rich stem anatomy functions as a hydraulic capacitor (Holbrook and Sinclair 1992; Sperling et al. 2015; Aparecido et al. 2015). However, even within dicotyledonous trees, we see a variation within the diurnal amplitude of stem water discharge, with *P. tenuifolium*, which displays the lowest wood density and the largest diurnal amplitude among dicotyledonous trees (Fig. 1). Species-level differences in wood anatomy, such sapwood/heartwood ratio or wood density and related parenchyma fraction, might explain such variation, including hydraulic capacitance (Richards et al. 2014). However, the role of parenchyma as a buffer to drought impacts and the possible role of this tissue for embolism repair is not commonly assessed. We highlight this knowledge gap for future studies and the use of palms as excellent model organisms (Klein et al. 2018; Ny et al. 2021; Kawai et al. 2022). We recommend including regular measures of leaf water potential and sap flow (Renninger et al. 2010; Aparecido et al. 2015), which would pave the way for a detailed understanding of hydraulic capacitance and conductance in this physiologically understudied group.

The critical stem RWC thresholds identified in Fig. 2 reveal a striking physiological divergence in terms of hydraulic function when exposed to drought stress between dicotyledonous trees and monocotyledonous palms: the latter maintain high diurnal discharge capacity at hydration levels where the former already exhibit significant impairment. This suggests that these palms have tolerance mechanisms allowing for continued stomatal opening and hydraulic function under lower relative tissue hydration—potentially linked to their unique vascular architecture (Renninger and Phillips 2016). Thus, palms can be positioned in the drought-avoidance hydraulic strategy group, which is less often described for non-deciduous trees (Oliveira et al. 2021).

Our study thus demonstrates greater seasonal and diurnal water storage capacity that allows these monocots to maintain transpiration through large internal storage release, buffering them from water deficits. Interestingly, despite experiencing strong physiological strain towards the end of the drought (Fig. 2), species like *A. vulgare* are often successful in disturbed or secondary environments, indicating they may operate with broader hydraulic fluctuations or greater stem capacitance that enable survival under variable hydration conditions. Previous work that included palms in modelling frameworks of eco-hydrological processes suggests that these plants have a significantly higher transpiration rate compared to trees with a similar diameter (Kunert et al. 2015). This is consistent with our data suggesting that palms can indeed mobilise larger quantities of water from their stems than dicots, especially when soil water becomes limiting. This additional hydraulic capacity could help explain the previously observed drought resistance in some Amazonian palms (Sousa et al. 2020; Rodrigues-Filho et al. 2024). Future studies should elucidate the contribution of these monocots to overall forest stand transpiration during drought (Renninger et al. 2010; Kunert et al. 2015; Brum et al. 2021).

Furthermore, our GAM analysis demonstrates threshold-like behaviour to soil drought as previously shown to be general for tree mortality and available soil moisture across different Amazon forests (Meir et al. 2015). Our data therefore hint at the possibility of a coordinated hydraulic response between soil water availability and internal stem water storage depletion, meaning that stem water reserves become rapidly depleted after crossing a certain soil moisture threshold. A recent study has identified soil moisture thresholds controlling transpiration in an Amazonian forest. In particular, sap flow responses from multiple species indicate a critical threshold in soil moisture at approximately 0.32 m³ m⁻³ (at ~ 0.5 m depth), below which transpiration declines rapidly (Chambers et al. 2025). Consistent with these findings, the soil drought of 2023 caused soil water content to fall continuously below 0.32 m^3^ m^− 3^ during the dry season 2023 in Caxiuanã (see Fig. 3), consistent with large reductions in transpiration (deviation from optima) measured at that site during that time (Martius et al. 2025). Chambers et al. (2025) also demonstrate that transpiration becomes severely limited at soil water content < 0.22 m^3^ m^− 3^. Interestingly, the data from this study (Fig. 3) show that stem water stores start to become depleted at soil water availability of ~ 0.22 m^3^ m^− 3^ (at 1 m depth), indicating that daily discharge might be larger than what can be refilled overnight. We show that this depletion of stem water content is non-linear and follows a threshold response. This might indicate that all the measured trees from our study may lose the ability to refill their stems once soil moisture drops below a threshold of approximately 0.19 m^3^ m^− 3^, leading to rapid depletion of internal stem water reserves. Interestingly, the soil moisture threshold identified here closely aligns with findings from a global synthesis, which reported an average critical soil moisture threshold of 0.19 m³ m⁻³ below which plant evapotranspiration becomes increasingly constrained, consistent with the depletion of stem water reserves observed in this study (Fu et al. 2024). This might reflect the point of hydraulic disconnection between the soil and the stem, where insufficient soil water availability prevents effective water uptake, forcing trees to rely more heavily/or entirely on stored water during periods of low soil moisture (Fig. 3). The steep drop in stem RWC after that threshold can be explained by the continued water discharge from the stem during the day (see Fig. 2), coupled with restricted water supply from the soil leading to no refilling of the stem. These findings give further evidence for the importance of biophysical threshold-responses from tissue to ecosystem scale (Meir et al. 2015; Binks et al. 2024; Fu et al. 2024; Chambers et al. 2025). Future work with larger datasets is needed to explore potential time-lagged and hysteresis responses during recovery (Brum et al. 2026).

Furthermore, we suggest that stem water mobilisation also follows a threshold-like behaviour (see Fig. 2), where a critically low amount of water in the plant induces turgor pressure loss in the guard cells, triggering strong stomatal closure responses. These findings highlight the importance of water availability through large pools in the stem for responses related to stomatal closure and productivity under soil drought (Meir et al. 2015; Martinez-Vilalta et al. 2019; Liu et al. 2025), especially in arborescent palms considering that these are non-deciduous but are also found within highly arid environments (Sperling et al. 2015; Du et al. 2025).

Our results strongly suggest that Amazonian palms display marked hydraulic strategies that are distinct from those of dicots. Interestingly, the high stem water content presented here and measured in Amazonian palms and additional Atlantic rainforest data (Fig. S2) align with values reported for date palms (*Phoenix dactylifera* L.) in arid environments (Sperling et al. 2015). Although palms form a key component of tropical forests, our understanding of palm responses to intensifying climate extremes in Amazonia remains very limited (Salm et al. 2015; Aparecido et al. 2015; Sousa et al. 2020; Amaral et al. 2025). Looking ahead, it will be essential to consider the role of water table depth in shaping palm drought responses, especially given their prevalence in shallow water table regions (Sousa et al. 2020; Binks et al. 2024). Recent research highlights that forests with a shallow water table might be resilient to moderate drought, but may be vulnerable to severe drought. (Muscarella et al. 2020; Esteban et al. 2021; Costa et al. 2023a). We would also like to highlight the need for a more comprehensive separation of atmospheric- from soil effects, and additional direct measurements of stomatal conductance in palms alongside diurnal stem water depletion to validate our assumptions (Mencuccini et al. 2024). Future research should examine species-level vulnerability curves and refill capacity to better predict palm performance under projected climate scenarios, particularly as El Niño events may become more frequent and severe (Sousa et al. 2020; Espinoza et al. 2024).

The FDR technology proved highly effective for quantifying water stress dynamics and detecting critical thresholds. Figure 1 demonstrates the sensors’ sensitivity to precipitation events, capturing immediate stem rehydration responses that validate their utility for drought monitoring (Martius et al. 2024). This technological approach enables precise identification of species-level thresholds and provides unprecedented temporal resolution for understanding hydraulic regulation under varying water availability. The demonstrated FDR methodology opens new avenues for studying palm and tropical tree hydraulics and their role in tropical forest water balance under changing precipitation patterns.

## Conclusion

Our findings suggest that palms display vastly different hydraulic strategies and water use dynamics due to their unique capacity to store and mobilise large quantities of water in their stems compared to co-occurring dicotyledonous trees. This hydraulic strategy positions palms as drought-avoidant species with notable resistance to short-term water deficits. Given that palms represent seven out of the twenty most abundant tree species across Amazonia, and thus disproportionally contribute to Amazonian forest basal area, their distinct water dynamics likely influence ecosystem-scale responses to climate extremes. However, despite their ecological and socio-economic importance, this significant and highly abundant group of plants remains vastly ignored in eco-physiological and ecological studies. The successful application of FDR technology provides a powerful tool for ongoing, high-resolution monitoring of palm water dynamics and drought responses. We strongly encourage future research to give a more detailed focus on palms to better understand their hydraulic strategies and responses to drought and their critical role in affecting tropical forest resilience under changing climate conditions.

## Supplementary Information

Below is the link to the electronic supplementary material.

Supplementary Material 1

## Data Availability

The data that support the findings of this study will be made available on GitHub and will be accessible at https://github.com/lionmartius/Palmae_Martii

## References

[CR1] Amaral MRM, DeArmond D, Marra DM et al (2025) Silt and sand are opposite predictors of Amazonian palm distribution. For Ecol Manag 598:123241. 10.1016/j.foreco.2025.123241

[CR2] Aparecido LMT, dos Santos J, Higuchi N, Kunert N (2015) Ecological applications of differences in the hydraulic efficiency of palms and broad-leaved trees. Trees 29:1431–1445. 10.1007/s00468-015-1223-2

[CR3] Aparecido LMT, dos Santos J, Higuchi N, Kunert N (2019) Relevance of wood anatomy and size of Amazonian trees in the determination and allometry of sapwood area. Acta Amazonica 49:1–10. 10.1590/1809-4392201800961

[CR4] Binks O, Meir P, Konings AG et al (2024) A theoretical framework to quantify ecosystem pressure-volume relationships. Glob Change Biol 30:e17567. 10.1111/gcb.17567 10.1111/gcb.17567 39501460

[CR5] Brum M, Deslauriers A, Vadeboncoeur M et al (2026) Employing a hysteresis approach to analyze shifts in tree physiological thresholds in response to drought. Plant Cell Environ n/a. 10.1111/pce.70498 10.1111/pce.70498 PMC13353555 41881845

[CR6] Brum M, Oliveira RS, López JG et al (2021) Effects of irrigation on oil palm transpiration during ENSO-induced drought in the Brazilian Eastern Amazon. Agric Water Manage 245:106569. 10.1016/j.agwat.2020.106569

[CR7] Buranelli C, Martius LR (2025) SteW ingredients: Site specific raw data for the global database of stem water measurements [Data set]. Zenodo. 10.5281/zenodo.17581211

[CR8] Cannell MGR (1984) Woody biomass of forest stands. For Ecol Manag 8:299–312. 10.1016/0378-1127(84)90062-8

[CR9] Chambers JQ, Nogueira Lima AJ, Pastorello G et al (2025) Hot droughts in the Amazon provide a window to a future hypertropical climate. Nature 1–7. 10.1038/s41586-025-09728-y 10.1038/s41586-025-09728-y 41372411

[CR10] Cooper DLM, Lewis SL, Sullivan MJP et al (2024) Consistent patterns of common species across tropical tree communities. Nature 1–10. 10.1038/s41586-023-06820-z 10.1038/s41586-023-06820-z PMC10808064 38200314

[CR11] Costa FRC, Schietti J, Stark SC, Smith MN (2023a) The other side of tropical forest drought: do shallow water table regions of Amazonia act as large-scale hydrological refugia from drought? New Phytol 237:714–733. 10.1111/nph.17914 35037253 10.1111/nph.17914

[CR12] Costa JA, Vellame LM, Costa CAG et al (2023b) Water storage of a typical tree species in the Caatinga biome (Caesalpinia pyramidalis Tul). Hydrol Process 37:e14970. 10.1002/hyp.14970

[CR13] Dalagnol R, Wagner FH, Emilio T et al (2022) Canopy palm cover across the Brazilian Amazon forests mapped with airborne LiDAR data and deep learning. Remote Sens Ecol Conserv 8:601–614. 10.1002/rse2.264

[CR14] Dargie GC, Lewis SL, Lawson IT et al (2017) Age, extent and carbon storage of the central Congo Basin peatland complex. Nature 542:86–90. 10.1038/nature21048 28077869 10.1038/nature21048

[CR15] de Lima TM, Portela RDCQ, Mendes ETB, Oda GA (2022) Species distribution modeling allied with land-use reveal priority sites and species for palm (Arecaceae) conservation in Rio de Janeiro, Brazil. Front For Glob Change 5: 10.3389/ffgc.2022.928446

[CR16] Du B, Franzisky BL, Muhammad W et al (2025) How to cope with stress in the desert—the date palm approach. Plant Cell Environ 48:768–780. 10.1111/pce.15188 39351860 10.1111/pce.15188 PMC11615422

[CR17] Eiserhardt WL, Svenning J-C, Kissling WD, Balslev H (2011) Geographical ecology of the palms (Arecaceae): determinants of diversity and distributions across spatial scales. Ann Bot 108:1391–1416. 10.1093/aob/mcr146 21712297 10.1093/aob/mcr146 PMC3219491

[CR18] Emilio T, Lamarque LJ, Torres-Ruiz JM et al (2019) Embolism resistance in petioles and leaflets of palms. Ann Botany 124:1173–1183. 10.1093/aob/mcz104 10.1093/aob/mcz104 PMC6943700 31227829

[CR19] EmilioT, Pereira H, Costa FRC (2021) Intraspecific variation on palm leaf traits of co-occurring species—Does local hydrology play a role? Front Forests Global Change 4:1–12. 10.3389/ffgc.2021.715266

[CR20] Emilio T, Quesada CA, Costa FRC et al (2014) Soil physical conditions limit palm and tree basal area in Amazonian forests. Plant Ecol Divers 7:215–229. 10.1080/17550874.2013.772257

[CR21] Espinoza J-C, Jimenez JC, Marengo JA et al (2024) The new record of drought and warmth in the Amazon in 2023 related to regional and global climatic features. Sci Rep 14:8107. 10.1038/s41598-024-58782-5 38582778 10.1038/s41598-024-58782-5 PMC10998876

[CR22] Esteban EJL, Castilho CV, Melgaço KL, Costa FRC (2021) The other side of droughts: wet extremes and topography as buffers of negative drought effects in an Amazonian forest. New Phytol 229:1995–2006. 10.1111/nph.17005 33048346 10.1111/nph.17005

[CR23] Fu Z, Ciais P, Wigneron J-P et al (2024) Global critical soil moisture thresholds of plant water stress. Nat Commun 15:4826. 10.1038/s41467-024-49244-7 38844502 10.1038/s41467-024-49244-7 PMC11156669

[CR24] Holbrook NM, Sinclair TR (1992) Water balance in the arborescent palm, Sabal palmetto. II. Transpiration and stem water storage. Plant Cell Environ 15:401–409. 10.1111/j.1365-3040.1992.tb00990.x

[CR25] Kawai K, Minagi K, Nakamura T et al (2022) Parenchyma underlies the interspecific variation of xylem hydraulics and carbon storage across 15 woody species on a subtropical island in Japan. Tree Physiol 42:337–350. 10.1093/TREEPHYS/TPAB100 34328187 10.1093/treephys/tpab100

[CR26] Klein T, Zeppel MJB, Anderegg WRL et al (2018) Xylem embolism refilling and resilience against drought-induced mortality in woody plants: processes and trade-offs. Ecol Res 33:839–855. 10.1007/s11284-018-1588-y

[CR27] Knauer J, El-Madany TS, Zaehle S, Migliavacca M (2018) Bigleaf—An R package for the calculation of physical and physiological ecosystem properties from eddy covariance data. PLoS ONE 13:e0201114. 10.1371/journal.pone.0201114 30106974 10.1371/journal.pone.0201114 PMC6091920

[CR28] Kunert N, Aparecido LMT, Barros P, Higuchi N (2015) Modeling potential impacts of planting palms or tree in small holder fruit plantations on ecohydrological processes in the central Amazon. Forests 6:2530–2544. 10.3390/f6082530

[CR29] Liu J, Wang Q, Zhan W et al (2025) When and where soil dryness matters to ecosystem photosynthesis. Nat Plants 1–11. 10.1038/s41477-025-02024-7 10.1038/s41477-025-02024-7 40624150

[CR30] Marengo JA, Espinoza J-C, Fu R et al (2024) Long-term variability, extremes and changes in temperature and hydrometeorology in the Amazon region: A review. Acta Amaz 54. e54es22098 10.1590/1809-4392202200980

[CR31] Martinez-Vilalta J, Anderegg WRL, Sapes G, Sala A (2019) Greater focus on water pools may improve our ability to understand and anticipate drought-induced mortality in plants. New Phytol 223:22–32. 10.1111/NPH.15644 30560995 10.1111/nph.15644

[CR32] Martins KKM, Vianna SA, Francisconi AF et al (2024) Neotropical palms: from their conservation to economic potential. Front Plant Sci 15. 10.3389/fpls.2024.1487297 10.3389/fpls.2024.1487297 PMC11620900 39649810

[CR64] Martius CFP von, Mohl H von, Unger F et al (1823) Historia Naturalis Palmarum. Opus Tripartitum. T.O. Weigel, Lipsiae. 10.5962/bhl.title.506

[CR33] Martius LR, Mencuccini M, Bittencourt PRL et al (2024) Towards accurate monitoring of water content in woody tissue across tropical forests and other biomes. Tree Physiol 44:tpae076. 10.1093/treephys/tpae076 38952005 10.1093/treephys/tpae076 PMC11299548

[CR34] Martius LR, Sanchez Martinez P, Negrão-Rodrigues V et al (2025) Amazônia doesn’t forget: Tropical trees with drought memory resist El Niño, EGU General Assembly 2025, Vienna, Austria, 27 Apr–2 May 2025, EGU25-3591. 10.5194/egusphere-egu25-3591

[CR36] Meir P, Wood TE, Galbraith DR et al (2015) Threshold responses to soil moisture deficit by trees and soil in tropical rain forests: Insights from field experiments. Bioscience 65:882–892. 10.1093/biosci/biv107 26955085 10.1093/biosci/biv107 PMC4777016

[CR37] Mencuccini M, Anderegg WRL, Binks O et al (2024) A new empirical framework to quantify the hydraulic effects of soil and atmospheric drivers on plant water status. Glob Change Biol 30:e17222. 10.1111/gcb.17222 10.1111/gcb.17222 38450813

[CR38] Muscarella R, Emilio T, Phillips OL et al (2020) The global abundance of tree palms. Glob Ecol Biogeogr 29:1495–1514. 10.1111/geb.13123

[CR39] Nakada R (2025) Factors influencing the moisture content of living tree stems measured by a dielectric soil moisture sensor. Bull FFPRI 24:57–77. 10.20756/ffpri.24.2_57

[CR40] Negrão-Rodrigues V, Bittencourt P, Sanchez-Martinez P et al (2025) Amazonian trees functional adjustments to long term experimental drought are limited and species dependent. Flora 331:152821. 10.1016/j.flora.2025.152821

[CR41] Ny A, Aritsara A, Razakandraibe VM et al (2021) Increasing axial parenchyma fraction in the Malagasy Magnoliids facilitated the co-optimisation of hydraulic efficiency and safety. New Phytol 229:1467–1480. 10.1111/nph.16969 32981106 10.1111/nph.16969

[CR42] Oda GA, Portela RCQ, Pires AS et al (2023) Distribution of leaflet traits across different habitats: a phylogenetically controlled test using Neotropical palms. Plant Ecol Divers 16:221–229. 10.1080/17550874.2023.2291044

[CR43] Olivares I, Svenning J-C, van Bodegom PM et al (2017) Stability in a changing world – palm community dynamics in the hyperdiverse western Amazon over 17 years. Glob Change Biol 23:1232–1239. 10.1111/gcb.13494 10.1111/gcb.13494 27614088

[CR44] Oliveira RS, Eller CB, de Barros F V, et al (2021) Linking plant hydraulics and the fast–slow continuum to understand resilience to drought in tropical ecosystems. New Phytol 230:904–923. 10.1111/nph.17266 33570772 10.1111/nph.17266

[CR45] Portela R, Silva V, Mendes E et al (2020) Differential phenological shifts in Euterpe edulis Mart. During an extremely dry year along an altitudinal gradient. Oecologia Australis 24:389–405. 10.4257/oeco.2020.2402.11

[CR63] R Core Team (2024) R: A language and environment for statistical computing. R Foundation for Statistical Computing, Vienna, Austria https://www.R-project.org/

[CR47] Reichgelt T, West CK, Greenwood DR (2018) The relation between global palm distribution and climate. Sci Rep 8:4721. 10.1038/s41598-018-23147-2 29549297 10.1038/s41598-018-23147-2 PMC5856843

[CR48] Renninger HJ, McCulloh KA, Phillips N (2013) A comparison of the hydraulic efficiency of a palm species (Iriartea deltoidea) with other wood types. Tree Physiol 33:152–160. 10.1093/TREEPHYS/TPS123 23296336 10.1093/treephys/tps123

[CR50] Renninger HJ, Phillips NG (2016) Palm physiology and distribution in response to global environmental change. In: Goldstein G, Santiago LS (eds) Tropical tree physiology: Adaptations and responses in a changing environment. Springer International Publishing, Cham, pp 67–101

[CR49] Renninger HJ, Phillips N, Salvucci GD (2010) Wet- vs. Dry-season transpiration in an Amazonian rain forest palm iriartea deltoidea. Biotropica 42:470–478. 10.1111/j.1744-7429.2009.00612.x

[CR51] Richards AE, Wright IJ, Lenz TI, Zanne AE (2014) Sapwood capacitance is greater in evergreen sclerophyll species growing in high compared to low-rainfall environments. Funct Ecol 28:734–744. 10.1111/1365-2435.12193

[CR52] Rodrigues-Filho CAS, Costa FRC, Schietti J et al (2024) Multi-taxa responses to climate change in the amazon forest. Glob Change Biol 30:e17598. 10.1111/gcb.17598 10.1111/gcb.17598 39605176

[CR53] Rosado BHP, Roland H, Moraes YCS (2023) On the biological concept of stress. Trends Ecol Evol 38:905–906. 10.1016/j.tree.2023.06.001 37365101 10.1016/j.tree.2023.06.001

[CR54] Salm R, Prates A, Simões NR, Feder L (2015) Palm community transitions along a topographic gradient from floodplain to terra firme in the eastern Amazon. Acta Amaz 45:65–74. 10.1590/1809-4392201401533

[CR55] Salomón RL, Limousin JM, Ourcival JM et al (2017) Stem hydraulic capacitance decreases with drought stress: implications for modelling tree hydraulics in the Mediterranean oak Quercus ilex. Plant Cell Environ 40:1379–1391. 10.1111/PCE.12928 28152583 10.1111/pce.12928

[CR56] Sanchez-Martinez P, Martius LR, Bittencourt P et al (2025) Amazon rainforest adjusts to long-term experimental drought. Nat Ecol Evol 1–10. 10.1038/s41559-025-02702-x 10.1038/s41559-025-02702-x PMC12148936 40374804

[CR58] Sousa TR, Schietti J, Coelho de Souza F et al (2020) Palms and trees resist extreme drought in Amazon forests with shallow water tables. J Ecol 108:2070–2082. 10.1111/1365-2745.13377

[CR59] Sperling O, Shapira O, Schwartz A, Lazarovitch N (2015) Direct in vivo evidence of immense stem water exploitation in irrigated date palms. J Exp Bot 66:333–338. 10.1093/jxb/eru421 25336690 10.1093/jxb/eru421 PMC4265166

[CR57] Słupianek A, Dolzblasz A, Sokołowska K (2021) Xylem Parenchyma—Role and relevance in wood functioning in trees. Plants 10:1247. 10.3390/plants10061247 34205276 10.3390/plants10061247 PMC8235782

[CR60] ter Steege H, Pitman NCA, Sabatier D et al (2013) Hyperdominance in the Amazonian Tree Flora. Science 342:1243092. 10.1126/science.1243092 24136971 10.1126/science.1243092

[CR61] Tomlinson PB (2006) The uniqueness of palms. Bot J Linn Soc 151:5–14. 10.1111/j.1095-8339.2006.00520.x

[CR62] Verona LS, Zanne AE, Trumbore S et al (2026) Vast, overlooked peat, and organic soils in Brazil’s Cerrado: carbon storage, dynamics, and stability. New Phytologist n/a. 10.1111/nph.71027 10.1111/nph.71027 PMC13150300 41817364

