# Supplementary material for: Stem Water Storage Dynamics in Amazonian Palms and Dicotyledonous Trees
Source: Trop Plant Biol. 2026 May 22;19(1):22. doi: 10.1007/s12042-026-09478-9 (PMC13197269; doi:10.1007/s12042-026-09478-9)
Supplement: Supplementary file 1 — Supplementary Material 1 [file 12042_2026_9478_MOESM1_ESM.docx]

**Supplementary Information for the paper entitled:**

**Stem water storage dynamics in Amazonian palms and dicotyledonous trees**

***Tropical Plant Biology***

Lion R. Martius, Thaise Emilio, Thales Moreira de Lima, Pablo Sanchez-Martinez, Antonio C. L. da Costa, Maurizio Mencuccini, Patrick Meir


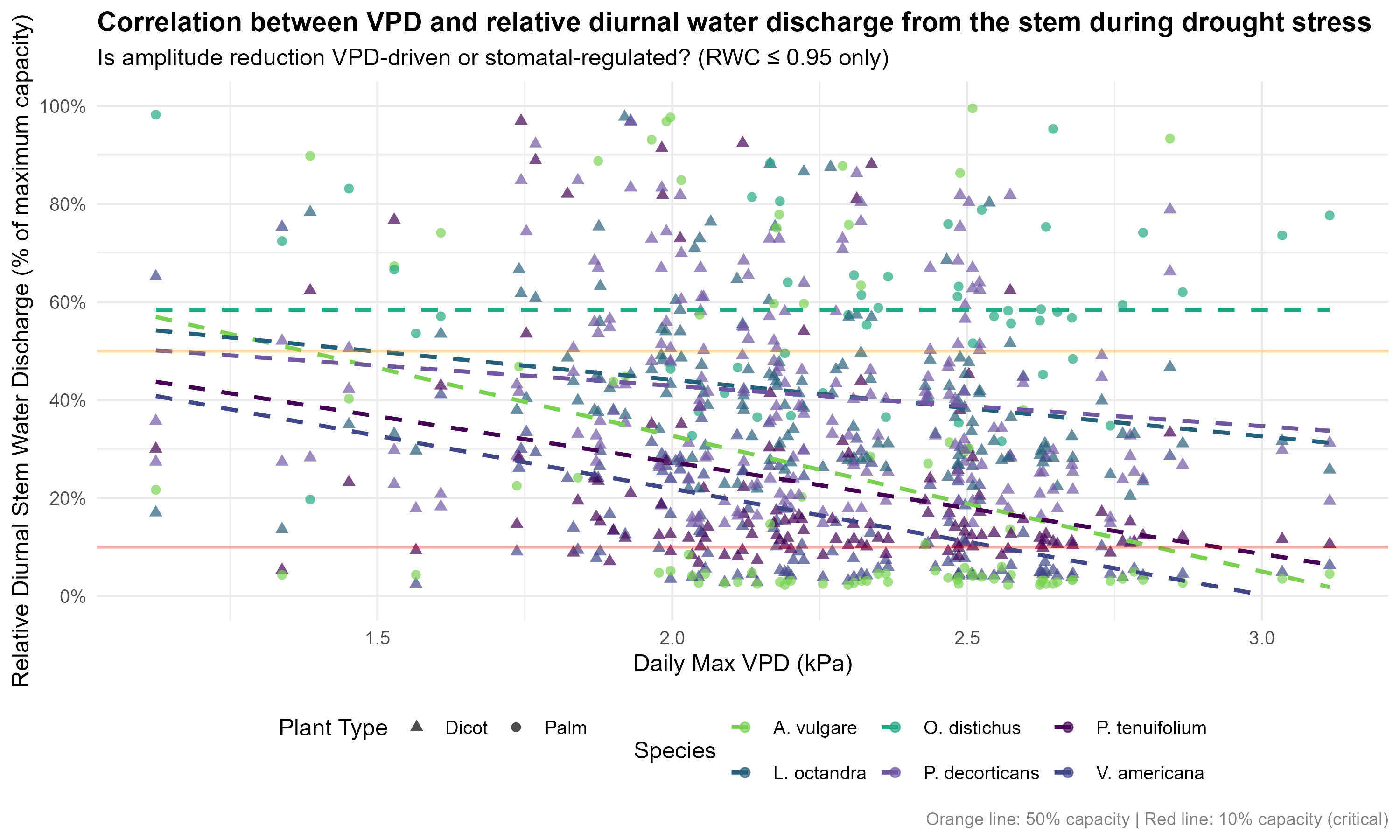


**Figure S1:** Relationship between daily maximum vapour pressure deficit (VPD) and relative diurnal amplitude during water stress conditions (RWC ≤ 0.95 only). Weak correlations between VPD and amplitude reduction across all species (palm mean r = -0.146 ± 0.159; dicot mean r = -0.297 ± 0.124) demonstrate that diurnal amplitude reductions during stress are primarily stomatal regulated, rather than driven by atmospheric demand. Points represent individual stress days coloured by species, with shapes indicating plant type (circles = palms, triangles = dicots). Dashed lines show linear trends for each species. Relative diurnal amplitude is expressed as percentage of tree specific maximum capacity, calculated using well-hydrated days (RWC > 0.95) as reference (95th percentile or maximum if <10 well-hydrated days available). Orange dashed line indicates 50% capacity threshold; red dashed line indicates 10% capacity (critical threshold). Data: n = 603 days across 7 individuals (2 palms, 5 dicots), July-December 2023. VPD represents daily 99^th^ percentile values.


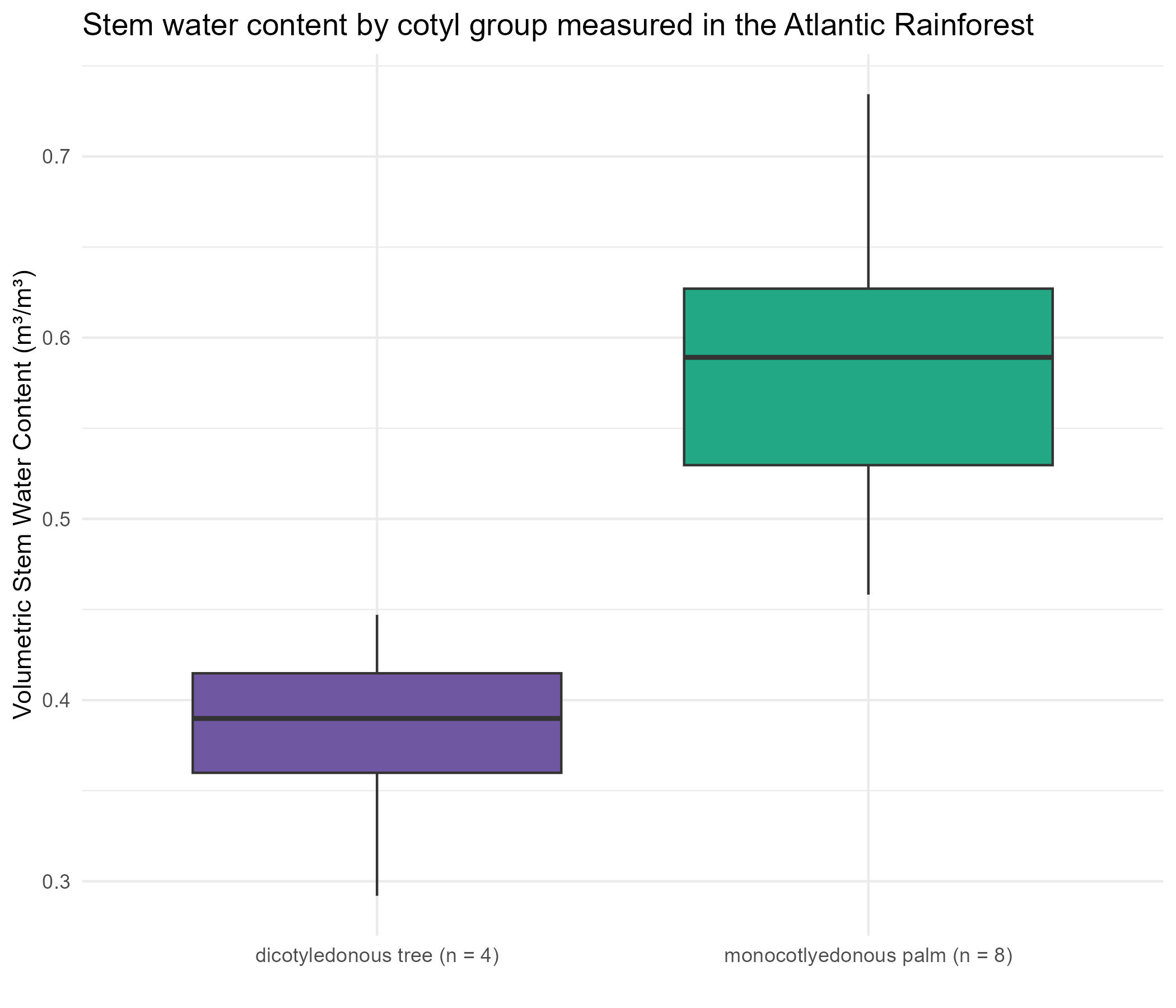


**Figure S2:** Median (horizontal line) and interquartile-range (extremes of the box) of the volumetric stem water content (m^3^ m^-3^) measured in eight palms (green; four Syagrus romanzoffiana (Cham.) Glassman and four Euterpe edulis Mart.) and four co-occurring dicotyledonous trees (purple; Protium spp.) in the Atlantic forest (Mata Atlântica) in the State of São Paulo, Brazil, between 05/11/2025 and 01/12/2025, using FDR sensors calibrated according to Martius et al. 2024. Palms demonstrated significantly larger stem water storage, with a mean volumetric water content of 0.59 ± 0.07 m^3^m^-3^ compared to dicotyledonous trees 0.38 ± 0.03 m^3^m^-3^ (t = 5.9, df = 10, p < 0.001). These quantitative differences in the stem water content between monocotyledonous palms and dicotyledonous trees in the Atlantic forest corroborate the results presented in this study on the differences between these plant groups in the Amazon rainforest.
